# Association of Combined Per- and Polyfluoroalkyl Substances and Metals with Chronic Kidney Disease

**DOI:** 10.3390/ijerph21040468

**Published:** 2024-04-11

**Authors:** Issah Haruna, Emmanuel Obeng-Gyasi

**Affiliations:** 1 Department of Built Environment, North Carolina A&T State University, Greensboro, NC 27411, USA; 2 Environmental Health and Disease Laboratory, North Carolina A&T State University, Greensboro, NC 27411, USA

**Keywords:** environmental pollutants, chronic kidney disease, metals, Bayesian kernel machine regression, NHANES, posterior inclusion probability, estimated glomerular filtration rate, PFAS, univariate and bivariate exposure–response

## Abstract

*Background*: Exposure to environmental pollutants such as metals and Per- and Polyfluoroalkyl Substances (PFAS) has become common and increasingly associated with a decrease in the estimated Glomerular Filtration Rate (eGFR), which is a marker often used to measure chronic kidney disease (CKD). However, there are limited studies involving the use of both eGFR and the urine albumin creatinine ratio (uACR), which are more comprehensive markers to determine the presence of CKD and the complexity of pollutant exposures and response interactions, especially for combined metals and PFAS, which has not been comprehensively elucidated. *Objective*: This study aims to assess the individual and combined effects of perfluorooctanoic acid (PFOA), perfluorooctanesulfonic acid (PFOS), Cadmium (Cd), Mercury (Hg), and Lead (Pb) exposure on CKD using data from the National Health and Nutritional Examination Survey (NHANES) 2017–2018. *Methods*: We employed the use of bivariate logistic regression and Bayesian Kernel Machine Regression (BKMR) in our analysis of the data. *Results*: Logistic regression results revealed a positive association between PFOA and CKD. Our BKMR analysis revealed a non-linear and bi-phasic relationship between the metal exposures and CKD. In our univariate exposure–response function plot, Cd and Hg exhibited a U and N-shaped interaction, which indicated a non-linear and non-additive relationship with both low and high exposures associated with CKD. In addition, the bivariate exposure–response function between two exposures in a mixture revealed that Cd had a U-shaped relationship with CKD at different quantiles of Pb, Hg, PFOA, and PFOS, indicating that both low and high levels of Cd is associated with CKD, implying a non-linear and complex biological interaction. Hg’s interaction plot demonstrated a N-shaped association across all quantiles of Cd, with the 75th quantile of Pb and the 50th and 75th quantiles of PFOA and PFOS. Furthermore, the PIP results underscored Cd’s consistent association with CKD (PIP = 1.000) followed by Hg’s (PIP = 0.9984), then PFOA and PFOS with a closely related PIP of 0.7880 and 0.7604, respectively, and finally Pb (PIP = 0.6940), contributing the least among the five environmental pollutants on CKD, though significant. *Conclusions*: Our findings revealed that exposure to environmental pollutants, particularly Hg and Cd, are associated with CKD. These findings highlight the need for public health interventions and strategies to mitigate the cumulative effect of PFAS and metal exposure and elucidate the significance of utilizing advanced statistical methods and tools to understand the impact of environmental pollutants on human health. Further research is needed to understand the mechanistic pathways of PFAS and metal-induced kidney injury and CKD, and longitudinal studies are required to ascertain the long-term impact of these environmental exposures.

## 1. Introduction

### 1.1. Chronic Kidney Disease

Chronic kidney disease (CKD) is a leading cause of morbidity and mortality and a significant public health concern globally. CKD is characterized by renal impairment, which is typically identified by specific markers such as albuminuria or an estimated glomerular filtration rate (eGFR < 60 mL/min/1.73 m^2^) for a minimum of three months, regardless of the underlying etiology [1,2,3].

Relevant data from the Global Burden of Disease (GBD) Chronic Kidney Disease Collaboration indicates that there were an estimated 697.5 million cases of CKD globally in 2017; as the population has aged, this number has increased and become a global public health concern [4,5]. According to a different report, the number of people with CKD has been rising, with an estimated 843.6 million people affected globally in 2017 [6,7].

According to the Centers for Disease Control and Prevention (CDC), CKD affects an estimated 35.5 million adults in the US alone (15% of the adult population; more than 1 in 7 adults), with a slightly higher prevalence in females (14%) than in males (12%). Those 65 years of age or older have a higher prevalence of CKD (34%) compared to those 45–64 years (12%) or 18–44 years old (6%). Non-Hispanic Black adults have a higher prevalence of CKD (20%) compared to non-Hispanic Asian adults (14%) or non-Hispanic white adults (12%). Approximately 14% of Hispanic adults have CKD, and as many as 9 in 10 adults do not know they have CKD [8].

Albuminuria (or proteinuria) and eGFR are typical markers of kidney damage. Albumin levels in 24 h urine (>30 mg) or urine sample albumin levels adjusted by urinary creatinine (>30 mg/g) are indicators of albuminuria. Thus, three levels of albuminuria are distinguished: normal (<30 mg/g), moderate (30–300 mg/g), and severe (>300 mg/g) [9]. CKD develops gradually; in the early stages, the kidneys can still filter waste, but in the latter stages, the kidneys either completely fail to function or lose most of their capacity. Consequently, there are five phases of CKD: stage 1 (mild kidney damage, eGFR ≥ 90), stage 2 (mild kidney damage, eGFR 60–89), stage 3 (moderate kidney damage, eGFR 30 and 59), stage 4 (moderate/severe kidney damage, eGFR 15–29), and stage 5 (kidney failure, eGFR ≤ 15) [3,10]. CKD emerged as the 18th most common cause of disability-adjusted life years (DALYs) worldwide in 2019 and is responsible for around 2% of all DALYs worldwide. Between 1990 and 2019, the number of DALYs related to CKD grew by 93%, from 21.5 million to 41.5 million [11], and it is anticipated that by 2030, it will rank as the thirteenth (13th) most common cause of death [12] and the fifth (5th) most common cause of death by 2040 globally [13].

### 1.2. Per- and Polyfluoroalkyl Substances (PFASs)

Per- and Polyfluoroalkyl substances are ubiquitous, man-made chemicals with a wide range of applications in industrial, household, and consumer products. They are used in the production of lubricants, paints, food packaging, fire-extinguishing foams, non-stick cookware coatings, and more [14,15,16].

PFASs are ubiquitously detected in human populations and many also have long elimination half-lives in humans; for instance, perfluorooctanesulfonic acid (PFOS) and perfluorooctanoic acid (PFOA) have half-lives of 3.4 years and 2.7 years, respectively [17]. Detectable quantities of specific PFASs were found in about 98% of blood samples taken from participants in the National Health and Nutrition Survey (NHANES) conducted in the United States [18,19].

Exposure to PFAS has been associated with obesity, hyperlipidemia, diabetes, and microvascular disease, which are conditions that are linked to poor kidney function [20]. Since the kidney is the primary route for PFAS elimination, continued exposure results in bioaccumulation may take several years to be eliminated [18,20]. Due to the long environmental persistence and biological half-lives of certain PFASs, exposure in food, water, and air has resulted in their detectable levels in the blood of almost all people in industrialized nations, with documented health impacts worldwide [21,22]. The extended persistence of long-chain PFASs within the human body is attributed to their active reabsorption by the renal tubules, which impedes their excretion and contributes to their prolonged half-lives in humans. Consequently, the presence of high concentrations of legacy PFAS, such as PFOA and PFOS, in kidney tissues is alarming. Indeed, studies focusing on histopathology, molecular oxidative stress, and epigenetics have revealed evidence suggesting the potential nephrotoxic effects of these compounds [23,24].

### 1.3. Metals and Nephrotoxicity

Environmental metals such as Cadmium (Cd), Lead (Pb), and Mercury (Hg) are among the top 10 most toxic substances of public health significance and have no established biological function in humans [25]. Heavy metals have been shown to have an impact on cellular organelles and components in biological systems, including the nucleus, mitochondria, lysosome, endoplasmic reticulum, and some enzymes that are involved in metabolism, detoxification, and the repair of damaged tissues [26]. The World Health Organization (WHO) has identified ten chemicals as environmental contaminants of serious public health concern, and Cd, Hg, and Pb are among them [27]. Most humans come into contact with these metals either through skin contact (through paint or soil), inhalation (through industrial products), or ingestion (through food and water) [28]. According to several studies, these heavy metals accumulate in the kidneys and can induce renal damage, proteinuria, and CKD even at low concentrations [29,30]. Chronic long-term exposure to Cd and Pb has been associated with distinct pathologies in nearly every tissue and organ throughout the human body [31]. In health risk assessments, the kidney was considered the critical target of Cd toxicity [31,32].

#### 1.3.1. Lead

Pb is a toxic metal whose widespread use has caused extreme environmental contamination and health problems in many parts of the world [33]. The causal link between exposure to Pb and significant cognitive health impacts is widely recognized, along with other consequences, including memory impairment, abdominal discomfort, renal injury, elevated blood pressure, and physical debility [34]. Previous research has demonstrated that minority and low-income communities bear the bulk of Pb exposure compared with their more affluent counterparts [35]. Children are particularly susceptible to the neurotoxic impacts of Pb, and exposure to even modest amounts can lead to severe and sometimes permanent damage to the nervous system [36].

The Earth’s crust contains trace amounts of Pb, mostly in the form of Pb sulfide (galena). However, the main cause of the ubiquitous presence of Pb in the environment is human activity, including Pb mining, smelting, refining, and recycling, the use of leaded gasoline and aviation fuel, and Pb in battery production, such as Pb-acid batteries [37]. Pb enters drinking water primarily through the corrosion of Pb-containing plumbing and fixtures [36]. Furthermore, Pb compounds were historically widely employed as pigment in paints (the paint in many homes was Pb-based, containing up to 40% Pb) and glazes for ceramics [29]. As reported by the Centers for Disease Control and Prevention, no level of Pb is safe in humans, especially in children, who can come into contact with Pb by touching, swallowing, or breathing in Pb or Pb dust [38].

#### 1.3.2. Cadmium

Chronic Cd poisoning, also known as the itai–itai disease, results in renal tubular failure, which was initially identified in Japan in the early 20th century and is characterized by osteomalacia and associated with renal tubular dysfunction [39,40]. Post-ingestion of Cd-contaminated water, food, or cigarette smoking, Cd can be absorbed into the bloodstream via the gastrointestinal tract, respiratory tract, or the skin, where it binds to albumin and other cysteine-containing proteins and peptides such as glutathione [41] and can cause the production of reactive oxygen species (ROS). Cadmium is a highly persistent environmental toxicant that is a food-chain pollutant of major public health concern since it demonstrates higher rates of soil-to-plant transfer than other toxic heavy metals like Pb and Hg [31].

#### 1.3.3. Mercury

Exposure to Hg compounds occurs via occupational, dietary, and environmental sources, including contaminated water, freshwater fish from a contaminated source, predatory ocean fish, gold mining, smelting, burning fuel, incineration, and various forms of whitening creams [29]. Chronic Hg poisoning can also result from the illicit use of cosmetics containing Hg compounds to whiten the skin, prevent facial plaque, and restrict the production of melanin [42]. The majority of individuals with Hg-induced kidney injury presented clinically with nephrotic syndrome, and the condition was more common in women [43]. Because of the strong affinity of Hg for kidney tissue, the kidney is one of the most susceptible organs to Hg poisoning [43]. When Hg accumulates in the body to a certain degree, kidney disease occurs and is reflected by proteinuria. Clinically, patients with renal damage secondary to Hg poisoning typically show edema, urine volume changes, proteinuria, and nephrotic syndrome [43,44]. 

### 1.4. Problem Statement

Humans are inevitably exposed to environmental pollutants, such as PFAS and heavy metals, simultaneously [45] and have been identified in many environmental media and human serum [46,47]. Exposure to drinking water is considered to contribute to the elevated amount in human serum [48], and other potential routes of human exposure include PFAS-contaminated food, food packaging, and PFAS-treated carpets, cookware (non-stick pans), electronics, and clothing [49,50]. Because of long half-lives of up to several years for PFOA as well as other long-chain perfluorinated substances such as PFOS, their levels observed in the general population mainly result from nutrition (like fish consumption), and other routes like dust ingestion and inhalation may also contribute to exposure [51]. In 2016, the United States Environmental Protection Agency (USEPA) published drinking water health advisories for combined or individual PFOA and PFOS concentration at 70 parts per trillion (ppt) [52]. The USEPA finalized its decision to regulate two contaminants, PFOS and PFOA, included in the Contaminant Candidate List 4 (CCL 4) for drinking water in the United States. The CCL 4 is curated by the USEPA to highlight contaminants that, although not yet regulated by national drinking water standards, are recognized for their presence in public water systems and might necessitate regulation under the Safe Drinking Water Act (SDWA) because of the health risks they pose. Following the conclusive regulatory decisions regarding PFOA and PFOS, the EPA plans to proceed with the process of developing national primary drinking water regulations for these two PFASs [53].

Several studies have looked at the detrimental effects of PFAS (such as PFOA and PFOS) and heavy metals on CKD individually [54,55], including recent epigenomic studies on the promotion of alterations in kidney tissues of mice from acute PFOA exposure [24] and the association of heavy metal exposure (including Pb, Cd, and Hg) on CKD [29,56,57]. Indeed, growing evidence has indicated that chronic exposure to environmental contaminants, such as heavy metals and persistent organic pollutants (POPs) like PFAS, may disrupt kidney function [24,58]. However, there are limited existing studies on the cumulative impact and the complex, non-linear, non-additive interaction of these environmental contaminants (PFAS and heavy metals) on CKD. Hence, there is a need to assess the association of combined PFAS and metals on CKD.

### 1.5. Significance of the Study

1. This study is aimed at providing an understanding of the environmental risk factors, particularly the combined effect of contaminants on CKD, which is one of the leading causes of death.
2. The present study also helps address the scarcity of studies exploring the combined effects of PFAS and metals on CKD.
3. The findings of this study may help regulatory bodies make policies for limiting exposure to these environmental pollutants and provide public health interventions.

### 1.6. Research Objectives

1. We aimed to explore the association between PFAS (PFOA and PFOS) exposure and CKD.
2. Examine the relationship between heavy metal (Lead, Cadmium, Mercury) exposure and CKD.
3. Investigate the potential synergistic effects of combined PFAS and metal exposure on CKD.

## 2. Materials and Methods

### 2.1. Study Population

This study used a subsample of the National Health and Nutrition Examination Survey (NHANES) 2017–2018, a multiphase, stratified study intended to offer a thorough analysis of the nutritional status and overall health of a nationally representative sample of non-institutionalized individuals in the USA. All participants provided informed consent, and the protocols were approved by the Institutional Review Board of the Centers for Disease Control and Prevention (CDC) at the National Center for Health Statistics (NCHS).

### 2.2. PFAS Extraction and Quantitation

The analytical technique employed for the quantitative detection of the PFAS was as follows: the sum of perfluoromethylheptane sulfonate isomers (Sm-PFOS, monomethyl branched isomers of PFOS), n-perfluorooctanoate (n-PFOA), n-perfluorooctane sulfonate (n-PFOS), ref. [59] and sum of branched perfluorooctanoate isomers (Sb-PFOA, branched PFOA isomers) forms the online solid phase extraction coupled to high-performance liquid chromatography–turboionspray ionization–tandem mass spectrometry (online SPE-HPLC-TIS-MS/MS). A 50 μL sample of serum was introduced into a commercially available column-switching system shortly after dilution with formic acid. This allowed the analytes to be concentrated on a solid-phase extraction column. Analytes were separated from other serum components and from each other using high-performance liquid chromatography. Detection and quantification were achieved using negative-ion TurboIonSpray ionization, a derivative of electrospray ionization, coupled with tandem mass spectrometry, offering sensitivity in the low parts per billion (ppb or ng/mL) range. This method facilitated the rapid detection of PFAS in human serum [60,61].

### 2.3. Plasma Heavy Metals (Lead, Cadmium and Mercury) Measurement

For the NHANES 2017–2018, metal assays were conducted on whole blood samples by the CDC’s Division of Laboratory Sciences at the National Center for Environmental Health using inductively coupled-plasma dynamic reaction cell-mass spectrometry (ICP-DRC-MS) to test the levels of heavy metals (Pb, Cd, and Hg) in blood. The NHANES website has detailed laboratory procedures regarding quality control and quality assurance data [62].

### 2.4. CKD Biomarkers

#### 2.4.1. Albuminuria (Urine Albumin)

The measurement of urinary albumin was conducted by a solid-phase fluorescent immunoassay (FIA) using the method described by [63]. This technique uses a double-antibody, solid-phase, non-competitive reaction. The albumin antigen in urine specimens reacts with the albumin antibody covalently bound to polyacrylamide beads. Then, the fluorescein-labeled antibody reacts with this solid-phase antibody complex. Centrifugation is used to extract additional proteins and unattached fluorescent antibodies. Using a fluorometer, one can detect the fluorescence of the stable solid-phase double-antibody complex, which is directly proportional to the concentration of urine albumin. The standard line calibration material is human serum albumin in the range of 0.5 to 20 μg/mL [64].

#### 2.4.2. Serum and Urine Creatinine

Serum and urine creatinine measurement in NHANES 2017–2018 was conducted by the Advanced Research and Diagnostic Laboratory (ARDL) at the University of Minnesota using the enzymatic method. In this enzymatic method, creatinine is converted to creatine under the activity of creatininase. Creatine is then acted upon by the enzyme creatinase to form sarcosine and urea. Arcosine oxidase catalyzes the conversion of sarcosine into glycine and hydrogen peroxide, which then reacts with a chromophore in the presence of peroxidase, leading to the formation of a colored substance. The absorbance of this substance is measured at a primary wavelength of 546 nm and a secondary wavelength of 700 nm. This endpoint reaction aligns closely with HPLC methodologies and offers a benefit over the Jaffe picric acid-based methods, which are prone to interference from non-creatinine chromogens [65,66].

#### 2.4.3. Urine Albumin-to-Creatinine Ratio

For the NHANES 2017–2018, one variable, URDACT (urine albumin/creatinine ratio) was created using the following formula: URDACT = URXUMA/URXUCR × 100, round to 0.01 [67]; in this context, URXUMA denotes urine albumin in micrograms per milliliter (μg/mL), and URXUCR indicates urine creatinine in milligrams per liter (mg/L). Albuminuria was classified based on the calculated albumin-to-creatinine ratios into two categories: microalbuminuria, ranging from 30 to 299 mg/g, and macroalbuminuria, exceeding 300 mg/g.

#### 2.4.4. Estimated Glomerular Filtration Rate 

The eGFR was determined through the use of the Modification of Diet in Renal Disease (MDRD) study equation [68,69]:

eGFR (mL/min/1.73 m^2^) = 175 × (Scr) − 1.154 × (Age) − 0.203 × (0.742 if female) × (1.210 if African American)

eGFR was categorized into five stages following the guidelines of the National Kidney Foundation [70,71]:

- Stage 1: eGFR is 90 mL/min/1.73 m^2^ or higher;
- Stage 2: eGFR ranges from 60 to 89 mL/min/1.73 m^2^;
- Stage 3: eGFR falls between 30 and 59 mL/min/1.73 m^2^;
- Stage 4: eGFR is within 15 to 29 mL/min/1.73 m^2^;
- Stage 5: eGFR is less than 15 mL/min/1.73 m m^2^.

#### 2.4.5. Chronic Kidney Disease 

CKD was categorized as either negative or positive, with positive encompassing all stages of CKD as per the classifications by the American Kidney Fund [72]:

- Stage 1: Persistent albuminuria with eGFR equal to or greater than 90 mL/min/1.73 m^2^;
- Stage 2: Persistent albuminuria with eGFR ranging from 60 to 89 mL/min/1.73 m^2^;
- Stage 3: eGFR between 30 and 59 mL/min/1.73 m^2^;
- Stage 4: eGFR between 15 and 29 mL/min/1.73 m^2^;
- Stage 5: eGFR less than 15 mL/min/1.73 m^2^.

CKD was further classified into three levels [71]:

- Negative: no CKD;
- Mild CKD: Stage 1 and 2;
- Moderate-to-severe CKD: Stages 3, 4, and 5.

### 2.5. Variables and Covariates for Model Adjustment

Our outcome variable was CKD, whereas the predictor variables (exposures) were PFAS (PFOA and PFOS) and metal exposure (Cd, Pb, and Hg). The covariates used for model adjustment included age, gender, race, smoking status, BMI, alcohol intake, diabetes, annual income, and hypertension. The potential covariates were selected according to previous studies on the effects of environmental pollutants such as metals on kidney health by [73,74].

### 2.6. Statistical Analysis

#### 2.6.1. Descriptive Statistics, Correlations, and Regression

Our study first involved the use of descriptive statistics to describe the exposure and demographic variables in the dataset. The Spearman and biserial Pearson correlation was used to assess the relationships among the pollutants and CKD. Logistic regression analysis was also conducted to determine their statistical significance. To ensure the integrity of our analysis, we imputed missing values within variables of interest using the median value. Our data analytic methodology began with data exploration and cleaning in which we addressed any discrepancies, missing records, or irrelevant information. This strategy helped to ensure that our study was based on complete datasets, limiting the possibility of bias caused by missing information.

#### 2.6.2. Bayesian Kernel Machine Regression (BKMR)

In this study, we used Bayesian Kernel Machine Regression (BKMR) utilizing the Markov Chain Monte-Carlo (MCMC) sampling approach, as described by Bobb et al. [75]. Our BKMR investigation produced Posterior Inclusion Probabilities (PIPs), which are crucial in determining the influence of individual PFASs and metals in a pollutant mixture, and the analytical process involved 5000 iterations. These PIPs, ranging from 0 to 1, aid in determining the relative importance of each metal and PFAS in the pollutant mixture [76]. To gain a better understanding of the interplay between these PFASs and metals and the outcome of interest (CKD), we calculated high-dimensional exposure–response functions, denoted as h(z), at various intervals. This was accomplished while holding the other impacting variables constant at their median [77].

The BKMR model stood out for its graphical interpretation function in our analysis. This capability enabled a comparative investigation of the impacts of metals and PFAS exposure, both collectively and individually, and compared the outcome observed at certain exposure percentiles to those at median exposure levels. It also emphasized the distinct association between each metal and PFAS and CKD, considering the constant median values of other exposures. This analytical technique enabled a greater understanding of the nuanced individual and cumulative effects of PFOA, PFOS, and metals such as Pb, Cd, and Hg on CKD.

Our study’s analysis was conducted with R (version 4.2.3; R Foundation for Statistical Computing, Vienna, Austria) [78]. The level of significance was set at 0.05 and adjusted for the covariates, BMI, gender, age, annual income, alcohol intake, smoking, and ethnicity.

## 3. Results

### 3.1. Demographics and Health Characteristics

The study (Table 1) encompassed a total of 9254 participants, exhibiting a relatively balanced distribution across genders, with 49.24% identified as male and 50.76% as female. The racial and ethnic composition of the participants was notably diverse, comprising 34.04% Non-Hispanic white, 22.85% Non-Hispanic Black, 14.77% Mexican American, 12.62% Non-Hispanic Asian, 8.86% Other Hispanic, and 6.85% Non-Hispanic Multiracial individuals. Furthermore, approximately 25.3% of the participants reported a household income below USD 25,000. Among these participants, 88.6% of participants admitted to alcohol consumption. Smoking was also prevalent, with 40.28% reporting to be smokers and 10.04% of participants responding that they had diabetes, a chronic medical condition. Furthermore, 4% reported experiencing weak or failing kidneys.

### 3.2. Kidney Biomarkers and CKD

The study (Table 1) also revealed a generally favorable kidney function, with an average eGFR of 98.40. A substantial majority, 86.66%, exhibited negative albuminuria, suggesting a low prevalence of early signs of kidney damage. Furthermore, 81.53% of participants did not have CKD, while 18.47% had CKD. Among those with CKD, 11.45% had mild CKD, and 8.83% had moderate-to-severe CKD, highlighting the varying degrees of disease severity. The most prevalent eGFR stages were Stage 1, constituting 55.04% of the population, and Stage 2, comprising 36.29%.

### 3.3. T-Test and Binary Logistic Regression Analysis

Table 2 presents the results of *t*-tests comparing the mean levels of environmental contaminants between individuals with chronic kidney disease (CKD) and those without CKD. For PFOS, the *p*-value of 0.0009 indicates a statistically significant difference in mean PFOS levels between individuals with CKD and those without CKD. This suggests that PFOS levels may be associated with the presence of CKD in the population under study. Similarly, for Pb and Cd, *p*-values < 0.0001 also indicated statistically significant differences in the mean levels between the two groups, suggesting potential associations with CKD. On the other hand, for PFOA and Hg, the *p*-values of 0.2848 and 0.5546, respectively, suggest no statistically significant differences in the mean levels between individuals with CKD and those without CKD, indicating no clear associations with CKD in this context.

The binary logistic regression analysis (Table 3) revealed that PFOS demonstrated a statistically significant inverse association with the outcome, with an odds ratio of 0.91 (95% CI: 0.79–1.01) and a *p*-value of 0.03. Conversely, PFOA showed a significant positive association with CKD, with an odds ratio of 1.68 (95% CI: 1.08–2.62), although this association was not statistically significant at the 0.05 significance level (*p* = 0.20).

For Pb, Hg, and Cd, our analysis did not reveal statistically significant associations with the outcome. The odds ratios were 1.53 (95% CI: 0.68–3.42), 0.69 (95% CI: 0.43–1.10), and 1.71 (95% CI: 0.49–5.98), respectively, with *p*-values of 0.28, 0.11, and 0.38.

**Figure 1:**
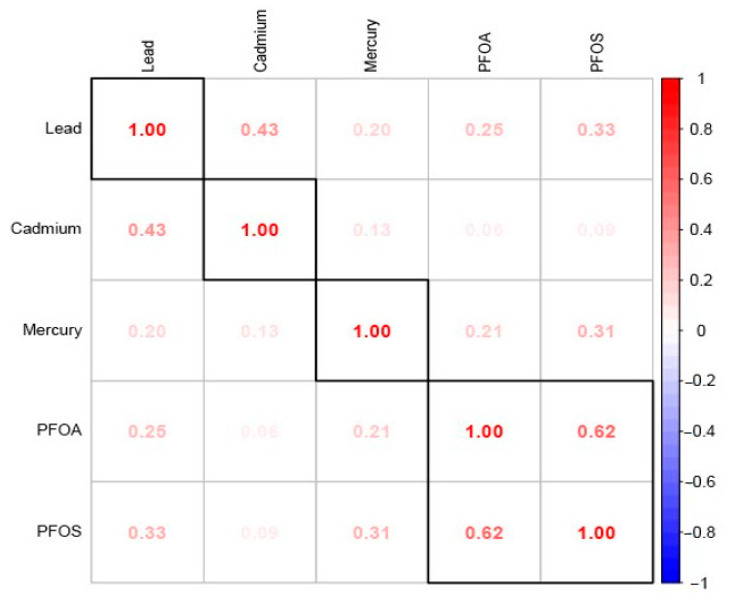
Spearman correlation among variables of interest.

Figure 1 displays the Spearman correlation analysis of the study’s exposure variables. The results show a strong positive relationship between the PFAS (between PFAS and PFOA), PFAS and metals, as well as among the metals themselves (inter-metals).

**Figure 2:**
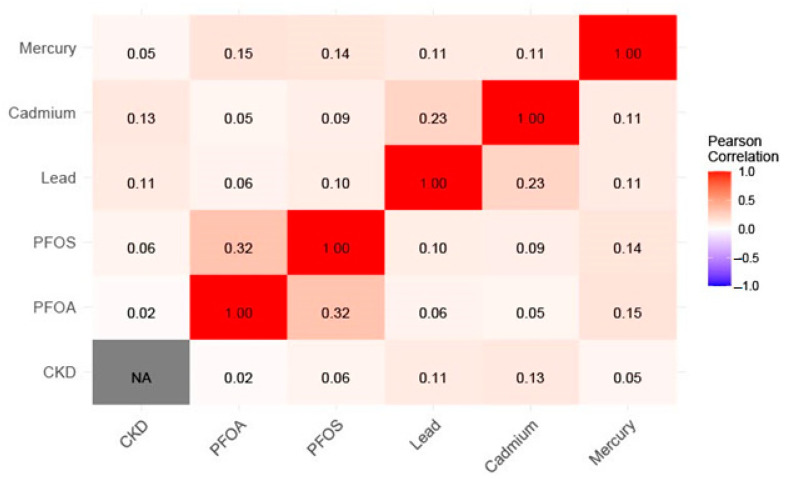
Pearson correlation matrix heatmap among outcome and exposure variables.

Figure 2 presents a biserial Pearson correlation matrix heatmap among outcome and exposure variables. The results reveal a positive correlation among the pollutant mixtures as well the between the pollutant and chronic kidney disease (CKD). The results reveal positive correlations among the PFAS (PFOA and PFOS) and metals (Pb, Hg, and Cd), indicating inter-pollutant correlations. Another evident correlation is the positive but weak correlation between Cd and Pb with CKD. Note: the red color legend represents a positive correlation, the grey area indicates no correlation, and the blue area represents a negative correlation (though not observed in this correlation heatmap).

### 3.4. BKMR

The statistically significant correlations found between the variables in our dataset indicated that BKMR analysis should be utilized instead of conventional regression techniques. The underlying premise of classic linear and logistic regression is the linearity of the relationship between the independent and dependent variables. These linear approaches, however, might not sufficiently capture the complex (non-linear and non-additive) nature of the data in real-world settings, where complex and potentially non-linear interactions exist among multiple environmental pollutants.

BKMR was able to assess relationships where complex and non-linear interactions between variables exist. Using Bayesian modeling and adjustable kernel functions, BKMR was able to consider interactions, capture complex dependencies, and reveal hidden patterns that logistic regression models might miss. This enabled more accurate, precise, and insightful findings.

#### 3.4.1. Quantifying the PFAS and Metal-Related Factors in CKD: PIP and BKMR Analysis

Table 4 presents the Posterior Inclusion Probability (PIP) for each PFAS and metal concerning its relationship with CDK. PIP in this analysis serves as a metric quantifying the likelihood of each contaminant playing a significant role in explaining their contribution to the presence of CKD. Cd stands out with the highest Probability of Inclusion (PIP) at 1.0000, marking it as the most strongly supported exposure for inclusion in the model and suggesting a highly likely connection with CKD. Following closely, Hg presents a PIP of 0.9984, which indicates a strong probable relationship with CKD. Meanwhile, PFOA and PFOS show moderate PIPs of 0.7880 and 0.7604, respectively, offering moderate support for their inclusion in the model and indicating reasonable but less significant links with CKD compared to Cd and Hg. Pb, despite being significant, possesses the lowest PIP of 0.6940 among the considered pollutants, which marks it as having the weakest evidence for inclusion and suggests a less probable link with the outcome compared to the other exposures.

Elevated PIP readings are suggestive of stronger evidence of association; therefore, based on the presented PIPs, Cd, and Hg, emerged as exposures with a higher probable relationship to CKD, followed by PFOS, PFOA, and Pb contributing the least, respectively. However, this study conducted additional statistical analysis to explore the non-linear and non-additive interactions among the pollutant mixtures and CKD.

#### 3.4.2. Univariate Analysis: Examining the Isolated Effects of PFOA, PFOS, Hg, Cd, and Pb on CKD

**Figure 3:**
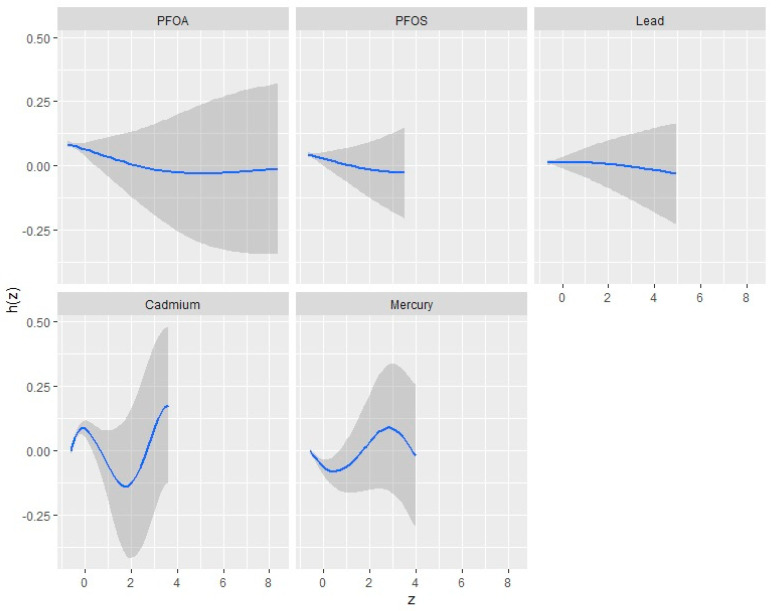
Univariate exposure–response functions and 95% confidence interval for the association between single pollutant exposure when other pollutant exposures are fixed at the median. Adjusted for age, gender, ethnicity, BMI, diabetes, annual income, alcohol intake, smoking, and hypertension.

The univariate approach visually examines the individual effects of PFOA, PFOS, Hg, Cd, and Pb on CKD. Figure 3 shows the impact of each pollutant on CKD when the others are fixed at the median and the covariates are held constant. The plot shows a suggestion of the non-linear effect of Cd and Hg.

With regard to Figure 3, the Pb and PFOS panel’s flat plot indicates that, over the range of exposures examined, differences in Pb exposure do not substantially impact CKD. This could indicate that Pb and PFOS are not significant contributors to CKD within the study’s observed exposure range or that other factors not included in this plot suppressed their influence.

The curve for PFOA decreases at higher exposure before plateauing, indicating that a decrease in PFOA exposure may be suggestive of lower risks of CKD initially. However, as its exposure decreases, the effect does not intensify, which indicates a threshold effect.

The U-shaped curve observed for the Cd and N-shape of Hg implies a non-linear relationship with CKD, implying that their low and high levels are associated with increased risk, whereas moderate levels are associated with a lower risk of CKD. In particular, the complex shape of Cd and Hg (U and N shape) is suggestive of a very complex non-monotonic relationship with CKD, which may indicate a complex non-additive biological effect or interactions between Cd, Hg, CKD. 

### 3.5. Visualizing Bivariate Exposure–Response Functions with Fixed Percentile Values

**Figure 4:**
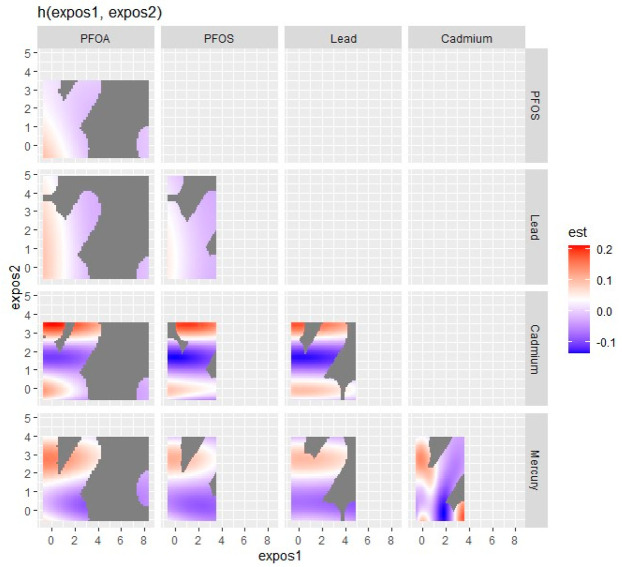
Bivariate exposure–response function of metals and PFAS with CKD.

The bivariate multipollutant exposure on CKD was investigated, with the effects of two of the pollutant mixtures of concern on CKD being assessed. At the same time, all other predictor variables were fixed at a specific percentile. The color scale (est) shows the estimated effect on CKD. In this graph (Figure 4), red denotes a greater positive effect (which increases the chance of the health outcome, CKD), blue shows a negative effect, and white or grey indicates no effect. 

The results observed in this figure suggest that the ‘PFOA’ vs. ‘PFOS’ plot (top left) indicates higher levels of both exposures, which seem to have a slight effect on CKD as indicated by the red region. A similar phenomenon can be observed in the ‘PFOA’ vs. ‘Lead’ and ‘PFOS vs. Lead’ plots. It can be seen that increasing levels of both ‘PFOA’ and ‘Cadmium’, ‘PFOS and Cadmium’ and particularly ‘Lead and Cadmium’ are associated with a stronger effect on the CKD, as indicated by the intense red area. This observation happens particularly with ‘PFOA and Mercury’, ‘PFOS and Mercury’, ‘Lead and Mercury’, and between ‘Cadmium and Mercury’ but to a more moderate extent (less intense redness). 

**Figure 5:**
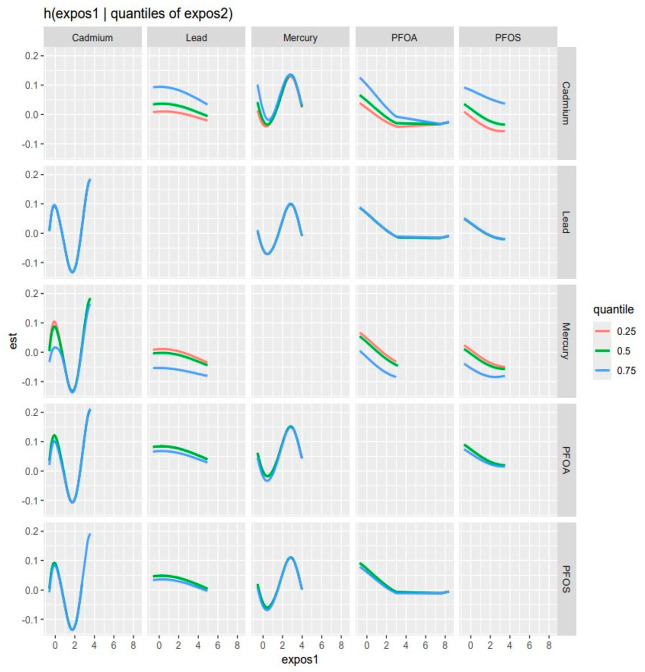
Bivariate exposure–response function of every two exposures with CKD—investigating the predictor-response function with varying quantiles of the second predictor, while other predictors are fixed. Adjusted for age, gender, ethnicity, BMI, diabetes, annual income, alcohol intake, smoking, and hypertension.

The bivariate association was subsequently investigated by looking at the pollutant pairs (Figure 5). The analysis investigated the relationship between specific pollutants and CKD by fixing the second pollutant at different quantiles of the 25th (red line), 50th (green line), and 75th (blue line) while keeping the other pollutant at the median. These models were adjusted for the covariates of interest (age, gender, BMI, income, ethnicity, diabetes, smoking, alcohol intake, and hypertension). The x-axis, designated “expos1”, depicts the levels of one exposure, whereas the y-axis, labeled “est”, displays the estimated effect on CKD. Each row of the graph represents a different exposure, denoted as “expos1”.

Interaction Effect: Each plot depicts how the relationship between “expos1” and CKD varies with the quantiles of a second exposure, “expos2”. The three lines within each plot represent the 25th, 50th, and 75th quantiles of “expos2”, as shown by the color legend.

The interpretation of the graph, as depicted by each pollutant, is as follows: 

Cadmium (as expos1): The plots for Cd reveal a U-shaped relationship with CKD at different quantiles of Pb, Hg, PFOA, and PFOS, indicating that both low and high levels of Cd are related to increased risk of CKD, implying a non-linear and complex biological interaction.

Lead (as expos1): Interacting with Pb, its effects observed on CKD appear relatively flat across all quantiles of Cd, Hg, and the 50th and 75th quantiles of PFOA and PFOS suggesting that lead’s effect on CKD is consistent regardless of the levels of Cd, Hg, PFOA, and PFOS.

Mercury (as expos1): Mercury’s interaction graphs reveal a strong and pronounced N-shape association with CKD across all the three quantiles of Cd, as well as a Pb (at 75th quantile), PFOA, and PFOS (at 50th and 75th). This implies that Mercury has a non-linear relationship with CKD levels, which could indicate a more nuanced interaction.

PFOA (as expos1): PFOA’s interaction graphs reveal an initial sharp decrease when interacting with Cd (at all quantiles), Pb (at the 50th quantile), Hg (at all quantiles), and PFOS (at the 50th and 75th quantile) and, later, a flat-shape (non-interactive) association with CKD at their respective Cd, Pb, and PFOS quantiles. This can be explained as the initial antagonistic effect of the interaction between PFOA and the other pollutants (except Hg) and the later non-interactive (flat shape) effect of PFOA on CKD regardless of an increase in levels of exposure particularly at or above point “3” on the X-axis (expos 1).

PFOS (as expos1): The PFOS interaction plot revealed a slight but not profound decreased association with CKD, indicating that low levels of Cd, Pb (at 75th quantile), Hg (across all quantiles), and PFOA (at 50th and 75th quantile) are related to a seemingly insignificant decreased effect on CKD. 

Influence of Quantiles: The changes in the shapes of the lines across different quantiles of “expos2” within each plot show how the influence of “expos1” on CKD varies with the “expos2” level. For example, in the bottom left plot (Cd interacting with PFOA and PFOS), the curves for the 50th and 75th quantiles of PFOA are nearly identical, indicating consistent effects at lower to mid-levels of Cd. However, at the 75th quantile, the curve rises more sharply, indicating the stronger interaction effect of PFOA and PFOS on CKD at higher Cd levels.

### 3.6. Overall Risk Summary of CKD with Exposure Percentiles

**Figure 6:**
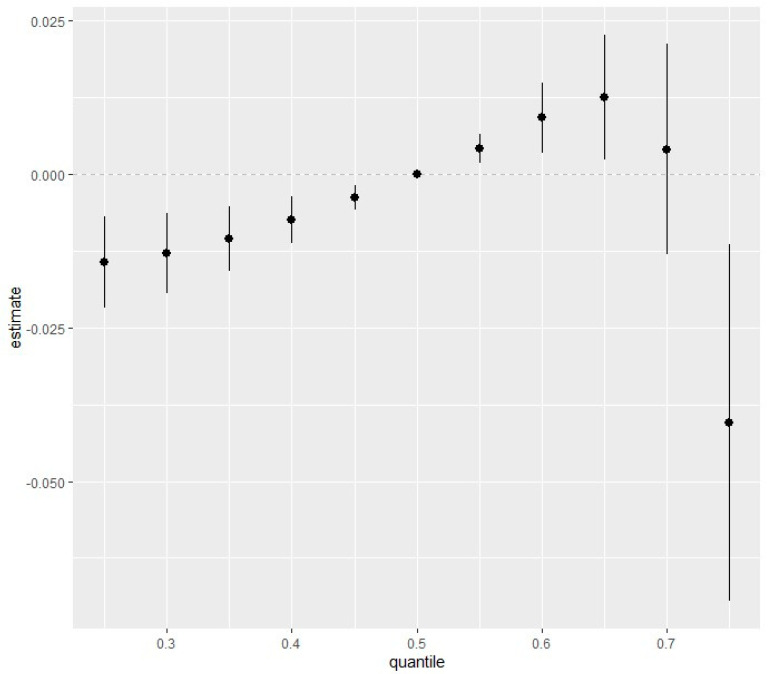
The summary of the overall health effects of the exposures (multiple pollutants) on the outcome depends on various percentiles (from 25th to 75th percentiles). Adjusted for age, gender, ethnicity, BMI, diabetes, annual income, alcohol intake, smoking and hypertension.

Figure 6 depicts the overall effect of all multipollutant mixtures on CKD. The exposures are fixed at different quantiles ranging from the 25th percentile to the 75th percentile in 5-point increments, with the 50th percentile (median value) serving as the point of comparison for the exposures. As shown in Figure 6, we found that the estimated risk of CKD increased with a simultaneous increase in all five pollutants, from the 25th percentile to the 65th percentile, compared to when all pollutants were at their 50th percentile (median values), indicating the positive joint effect of the pollutant mixtures. Particularly, when all five pollutants were within their 55th and 60th percentile, the estimated change in CKD was statistically significant (thus, its 95% credible interval did not overlap with zero). However, the overall effect began to decrease from the 65th to the 75th percentile, with less statistical significance (and the 95% confidence interval overlapping with and falling below zero).

### 3.7. Single-Variable Effects of PFAS and Metals on CKD

**Figure 7:**
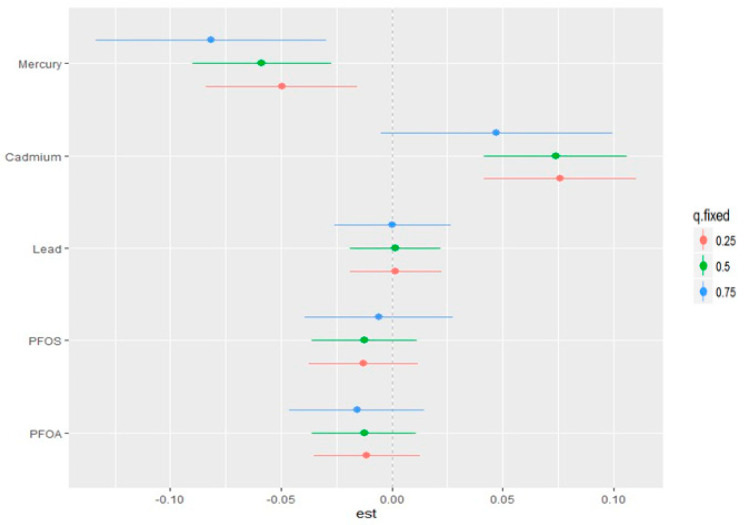
Single-variable effect of PFAS and metals at increasing quartiles for CDK. Adjusted for age, gender, ethnicity, BMI, diabetes, annual income, alcohol intake, smoking, and hypertension.

The single-variable effect aids in understanding the impact of a single predictor at various quantiles, allowing us to analyze their contribution to the total risk of CKD. Figure 7 depicts the single-variable effects of pollutants on CKD at the 25th (blue), 50th (green), and 75th (red) percentiles, which suggests that Cd is associated with higher values of the h function: a flexible function that takes multiple pollutants and combines them in a way that captures the complex and potentially non-linear relationship between the pollutants and CKD. Overall, the plot and quantiles demonstrate how the link between each pollutant and CKD varies depending on the exposure distribution of the pollutants.

## 4. Discussion

This study explored the subtle and nuanced relationships between exposure to various heavy metals (Cd, Pb, and Hg) and PFAS (PFOA and PFOS) on CKD, as quantified by kidney markers such as the eGFR and uACR, utilizing logistic regression and BKMR. Our results substantiate the proposed relationship between metals and PFAS-induced reduction in eGFR and increased uACR, which were used to determine CKD, drawing attention to the intricate interplay between Pb, Cd, Hg, PFOA, and PFOS and their combined influence on CKD.

Our study was able to reveal the complex non-linear and non-additive relationships and potential synergistic and antagonistic interactions among the array of metal and PFAS exposures, with interplays that remain hidden and masked with the use of conventional models alone. Cd, Hg, and PFOS (to a comparatively lesser extent), in particular, emerged with a pronounced non-linear relationship with CKD, which is a double-phase (diphasic) trend suggesting that both decreased and increased levels of their exposures significantly affect CKD.

The effects of Cd and Hg on nephrotoxicity and kidney disease have been explored in previous studies [79]. Again, among the environmental pollutants to which the general population is exposed throughout their lifetimes and that can have harmful effects on humans, specific attention has been given to toxic heavy metals, including, Pb, Hg, and Cd. Pb, Hg, and Cd, which were ranked second, third, and seventh, respectively, on the 2022 priority list of hazardous substances of the Agency for Toxic Substances and Disease Registry [80]. Studies from Yun et al. [81] found individual PFAS and their sum (including PFOA and PFOS) to be significantly associated with decreased eGFR (kidney function). The primary toxic effect of these environmental pollutants is the hypertrophic and histological changes they exert on the kidneys as a result of the increased production of reactive oxygen species (ROS), which results in changes in the permeability of microvascular endothelial cells, leading to a reduction in kidney performance [81,82]. These studies only relied on eGFR as a measure of kidney function. However, our findings utilized both eGFR and uACR, which are the two comprehensive markers for determining kidney function and CKD [70], and add to the nuance of how exposure to these multiple pollutants relates to CKD. This nuanced understanding has profound clinical implications, especially for populations with high environmental exposure, burden, signaling an increase in public health initiatives and interventions to consider the intricate and cumulative effects of PFAS and metal exposures.

To further explore these complexities, our study leveraged the Posterior Inclusion Probability as an analytical tool to measure the significance of each pollutant’s role in the observed variations in CKD outcome. Cd and Hg’s unequivocal PIP of 1.0000 and 0.9984 firmly established their consistent association with CKD as an indication of their significant influence on the disease condition (with Mercury having the highest contribution). PFOA and PFOS’s substantial PIP of 0.7880 and 0.7604, respectively, exert a similar contribution towards CKD along with lead’s above-average PIP of 0.6940, which depicts a more heterogeneous picture of influence, suggesting that their impact on kidney function may be modulated by a conflux of exposure levels, biological interactions [83], and other methodological nuances of the model.

Comparatively, our analysis of critical variables from the single pollutant effect shows that Cd had a statistically higher correlation with CKD, which affirms its differential impact on CKD. Though Hg did not exhibit the same direct effect, the U-shaped response plot in the univariate and bivariate exposure response analysis unveils a non-linear and potential synergism [84], signifying Pb and PFOA’S effect in a combined multipollutant mixture.

Cd and Hg exhibited a biphasic effect on CKD in both the univariate exposure–response and bivariate exposure–response function in our BKMR analysis, where their toxic effect initially decreased and then sharply rose, with the initial decrease in the toxic effect at lower doses followed by an increase in the toxic effect at higher doses. This non-linear dose–response relationship is characterized by the U-shaped plot.

This concept of biphasic effect challenges the traditional linear model of toxicology, where the dose–response relationship is assumed to be linear throughout. Instead, as unveiled by the BKMR model, it suggests that some level of exposure to a toxic substance at low doses may induce a decreased response. However, as the dose increases beyond a certain threshold, its harmful effect becomes dominant [85].

The outcome of the bivariate exposure–response functions further elucidates the potential for synergistic interactions among the pollutant mixtures, between metals [86] and PFAS [87], and between the metals (metal to metal) and the PFAS (PFAS to PFAS) [83], especially the dynamic interaction between Cd and Hg, between Pb and Cd, between PFOA and PFOS as well as between PFOS, Cd, and Hg. This synergism, which could magnify the risk of CKD, emphasizes the need for public health policies and interventions to mitigate the heterogenous and sophisticated risk of combined metal and PFAS exposures.

Overall, BKMR analysis offered significantly more insight into the relationship between combined metals and PFAS on CKD compared to logistic regression. For instance, the significant negative relationship shown by the logistic regression analysis for PFOA with CKD was not confirmed by the BKMR analysis, which rather helped to unmask the profound effects of Cd and Hg. This speaks to the need for using advanced techniques, such as BKMR, to comprehensively assess these relationships.

The implications of our findings are multifaceted, which advances beyond providing awareness and calling for public health intervention to inform future mechanistic research and further understand the complex biological interplay among pollutant mixtures and several chronic and malignant diseases. For instance, the attribution of our findings on metal- and PFAS-induced ROS, which leads to oxidative stress and eventually results in a reduction in kidney function and subsequent damage [81], could be further investigated. This important insight could be useful for the diagnosis of multipollutant-induced chronic disorders and cancers, given the prevalence of multiple pollutants in our environment [84]. Hence, our study’s disclosure of the subtle interaction and influence of combined metals and PFAS reveals the critical interplay of environmental factors and their influence on human diseases.

Overall, the use of advanced statistical methods and tools, such as BKMR, to ascertain the intricate intersectionality of environmental exposures to human diseases has become clear since traditional methods may not bring to light the non-linear, non-additive, and complex biological interactions.

### Limitations

Our study, though impactful, is not without its limitations. One major limitation of this study is its reliance on cross-sectional data from the NHANES survey, which hinders the ability to establish temporal or causal relationships between pollutant exposure and chronic kidney disease outcomes. Additionally, while this study examines multiple environmental pollutants, it may overlook potential interactions with other unmeasured variables, such as genetic predispositions or dietary factors, which could confound the observed associations. In future studies, enhancing the robustness of causal inference methodologies could be an area for improvement. While the current study utilizes advanced statistical methods, such as logistic regression and Bayesian Kernel Machine Regression, incorporating additional approaches, such as propensity score matching or instrumental variable analysis, could further strengthen causal inference. These methods could help address potential confounding factors and increase confidence in establishing causal relationships between environmental pollutant exposure and chronic kidney disease outcomes. To address this limitation and ascertain causation, there is a need for longitudinal studies or experimental designs. These approaches could track exposure over time and its direct impact on the development of CKD, thereby providing a more definitive understanding of these relationships.

## 5. Conclusions

This study broadens our understanding of the intricate interactions and mechanisms by which environmental PFASs and metals can influence the risk of CKD and potentially contribute to the etiology of chronic and malignant diseases. It underscores the utilization of BKMR in environmental health research and reinforces the usefulness of ongoing research into the long-term effects of combined PFASs and metal exposures. Our study supports a significant paradigm shift in the area of environmental pollutants and health, advocating for a holistic approach to exposure assessment and risk management to better improve and protect public health.

## Figures and Tables

**Table 1:** Descriptive statistics for critical variables of the study participants.

Variable	Mean (SD)	Minimum	25th Percentile	Median (50th)	75th Percentile	Maximum
PFOS	6.51 (7.74)	0.14	2.40	4.20	7.80	104.9
PFOA	1.71 (1.82)	0.14	0.87	1.37	2.07	52.87
Pb	1.08 (1.29)	0.05	0.46	0.76	1.30	42.48
Hg	1.14 (2.27)	0.20	0.20	0.51	1.12	63.64
Cd	0.37 (0.50)	0.07	0.12	0.22	0.42	13.03
Age	34.33 (25.50)	0	11	31	58	80
BMI	26.58 (8.26)	12.30	20.40	25.80	31.30	86.20
Kidney Biomarker						
Serum creatinine	0.88 (0.45)	0.25	0.68	0.82	0.98	12.74
Albumin creatinine ratio	42.80 (299.69)	0.27	5.23	8.28	15.88	11,676.92
eGFR	98.40 (36.41)	3.44	76.32	93.58	114.31	523.29
Variable	Description	Frequency (*n*)	percentage (%)			
Gender		9254				
	Male	4557	49.24			
	Female	4697	50.76			
Race/ethnicity	Mexican American	1367	14.77			
	Other Hispanic	820	8.86			
	Non-Hispanic White	3150	34.04			
	Non-Hispanic Black	2115	22.85			
	Non-Hispanic Asian	1168	12.62			
	Non-Hispanic Multiracial	634	6.85			
Alcohol use		5130				
	Yes	4545	88.6			
	No	585	11.4			
Smoking		5856				
	Yes	2359	40.28			
	No	3497	59.72			
Hypertension		1942				
	Yes	1650	84.96			
	No	292	15.04			
Having diabetes		8897				
	Yes	893	10.04			
	No	7816	87.85			
	Borderline	184	2.07			
	Don’t know	4	0.04			
Having weak/failing kidneys		5569				
	Yes	223	4.0			
	No	5337	95.83			
	Don’t know	9	0.16			
Variable	Description	Frequency (*n*)	Percentage (%)			
Albuminuria	Negative	6614	86.66			
	Microalbuminuria	864	11.32			
	Macroalbuminuria	154	2.02			
		5903				
eGFR stages	Stage 1	3249	55.04			
	Stage 2	2142	36.29			
	Stage 3	462	7.83			
	Stage 4	34	0.58			
	Stage 5	16	0.27			
CKD		5800				
	Negative	4729	81.53			
	positive	1071	18.47			
CKD Levels	Negative	4729	81.53			
	Mild	664	11.45			
	Moderate-to-severe	512	8.83			
CKD Stages	Negative	4729	80.08			
	Stage 1	383	6.49			
	Stage 2	281	4.76			
	Stage 3	462	7.82			
	Stage 4	34	0.58			
	Stage 5	16	0.27			

Note: PFOS: perfluorooctanesulfonic acid, PFOA: perfluorooctanoic acid, Pb: Lead, Hg: Mercury, Cd: Cadmium, BMI: Body Mass Index; and eGFR: estimated Glomerular Filtration Rate and CKD: chronic kidney disease.

**Table 2:** Statistical analysis of the association between the exposures (environmental pollutants) among CKD and non-CKD participants.

Variable	Mean (SD) *n* = 9254	CDK (+) *n* = 1071	No CDK (−) *n* = 4729	*p*-Value
PFOS	6.51 (7.74)	7.07 (8.93)	6.24 (7.44)	0.0009 *
PFOA	1.71 (1.82)	1.77 (1.24)	1.70 (1.93)	0.2848
Pb	1.08 (1.29)	1.35 (1.17)	1.09 (1.36)	<0.0001 *
Hg	1.14 (2.27)	1.13 (2.72)	1.32 (2.45)	0.5546
Cd	0.37 (0.50)	0.50 (0.62)	0.42 (0.52)	<0.0001 *

*p*-value is significant at <0.05 (* *p* < 0.05).

**Table 3:** Binary logistic regression analysis of exposure variables with CKD.

Variable	Odds Ratio (95% Confidence Interval)	*p*-Value
PFOS	0.91 (0.79–1.01)	0.03 *
PFOA	1.68 (1.08–2.62)	0.20
Pb	1.53 (0.68–3.42)	0.28
Hg	0.69 (0.43–1.10)	0.11
Cd	1.71 (0.49–5.98)	0.38

*p*-value is significant at <0.05 (* *p* < 0.05). Adjusted for age, gender, ethnicity, BMI, diabetes, annual income, alcohol intake, smoking and hypertension.

**Table 4:** Posterior Inclusion Probabilities for the influence of PFAS (PFOA and PFOS), Lead, Cadmium, and Mercury on chronic kidney disease (CKD).

Variable	PIP
PFOA	0.7880
PFOS	0.7604
Lead	0.6940
Cadmium	1.0000
Mercury	0.9984

Bayesian Posterior Inclusion Probabilities (PIPs) reveal candidate exposures associated with the outcome variable (CKD). Adjusted for age, gender, ethnicity, BMI, diabetes, annual income, alcohol intake, smoking, and hypertension.

## Data Availability

The NHANES dataset is publicly available online, accessible at https://www.cdc.gov/nchs/nhanes/.htm (accessed on 12 December 2023).
